# Supplementary material for: IgG sequencing maps systemic antibody binding to oral commensals and pathobionts in health and periodontitis
Source: bioRxiv. 2026 Oct 3:2026.09.28.753837. Preprint. [Version 1] doi: 10.64898/2026.09.28.753837 (PMC13648604; doi:10.64898/2026.09.28.753837)
Supplement: Supplementary file 1 [file NIHPP2026.09.28.753837v1-supplement-2.pdf]

bioRxiv preprint doi: https://doi.org/10.64898/2026.09.28.753837; this version posted October 3, 2026. The copyright holder for this preprint (which was not certified by peer review) is the author/funder. This article is a US Government work. It is not subject to copyright under 17 USC 105 and is also made available for use under a CC0 license.

Supplemental Table 1. Demographic characteristics of study participants and experiments performed for each individual.

Supplemental Table 2. Subject-wise Spearman correlation between relative abundance of Saliva and Gingival Crevicular Fluid (GCF).

Supplemental Table 3. GCF and Salivary IgG ICI scores for taxa highly coated in GCF.

Supplemental Table 4. Salivary and serum ICI scores by taxon in health and periodontitis.
