# Supplementary material for: IgG sequencing maps systemic antibody binding to oral commensals and pathobionts in health and periodontitis
Source: bioRxiv. 2026 Oct 3:2026.09.28.753837. Preprint. [Version 1] doi: 10.64898/2026.09.28.753837 (PMC13648604; doi:10.64898/2026.09.28.753837)
Supplement: Supplementary file 2 [file media-1.pdf]

A.

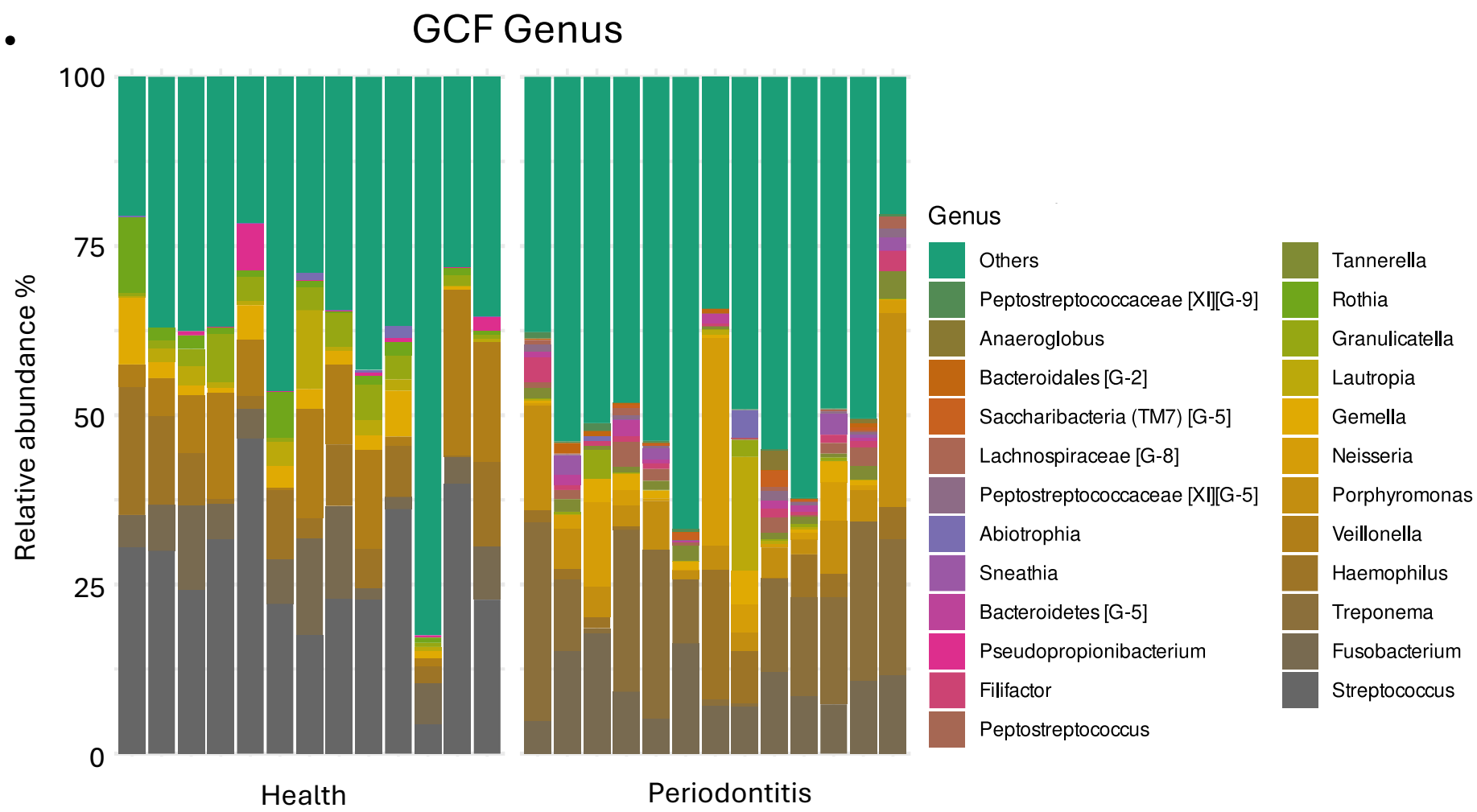

B.

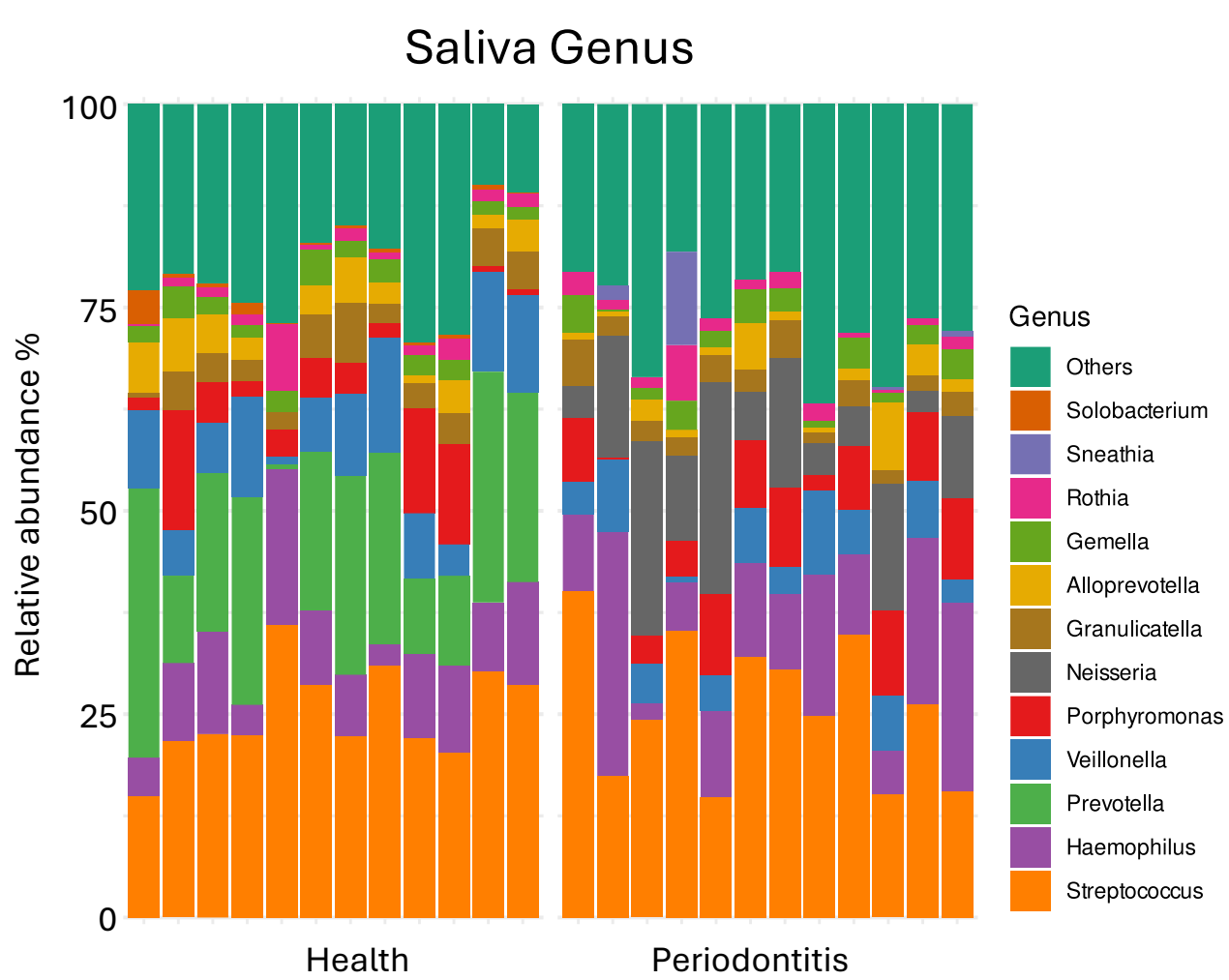

Suppl. Figure 1

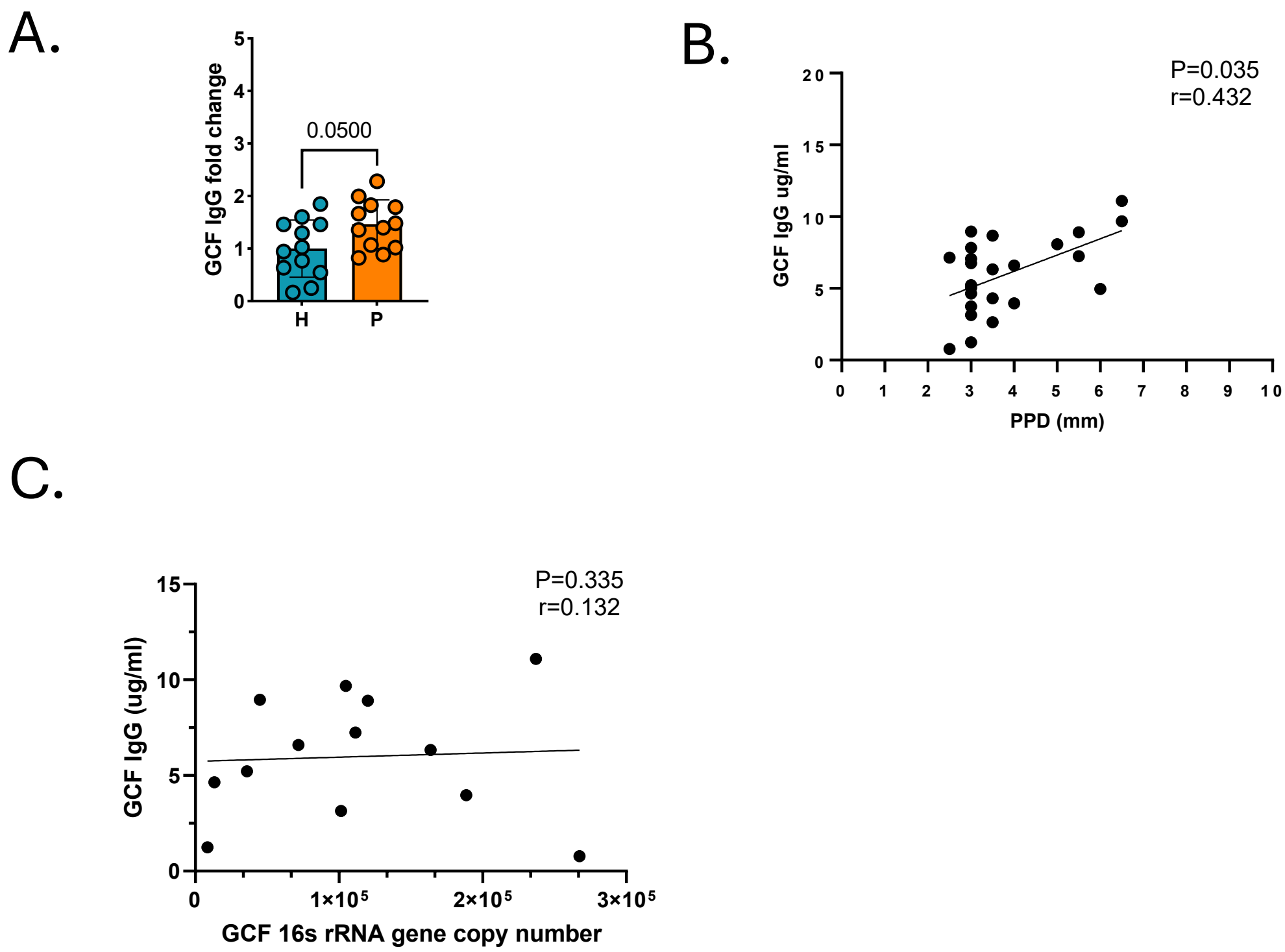

Suppl. Figure 2

A.

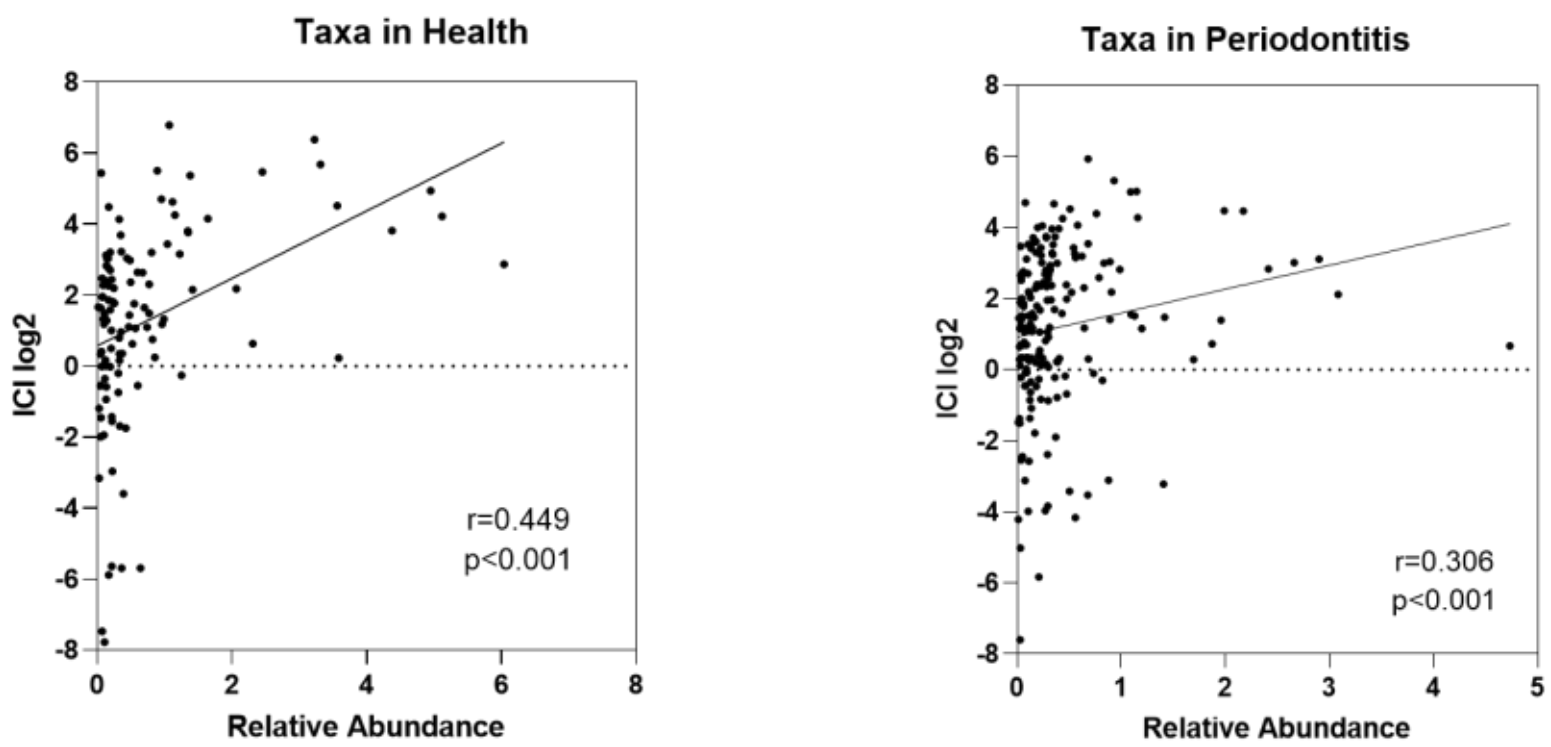

B.

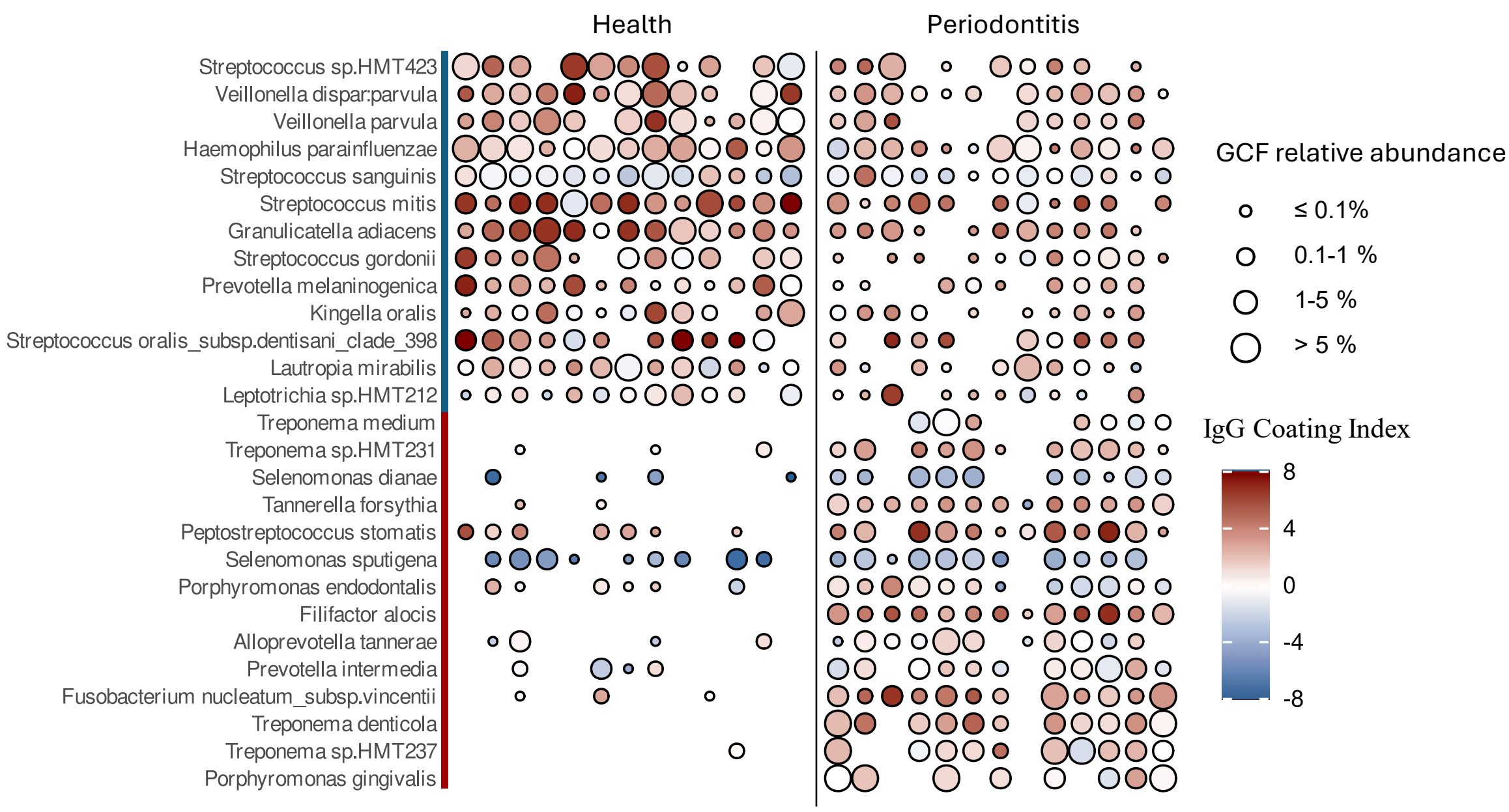

Suppl. Figure 3

A.

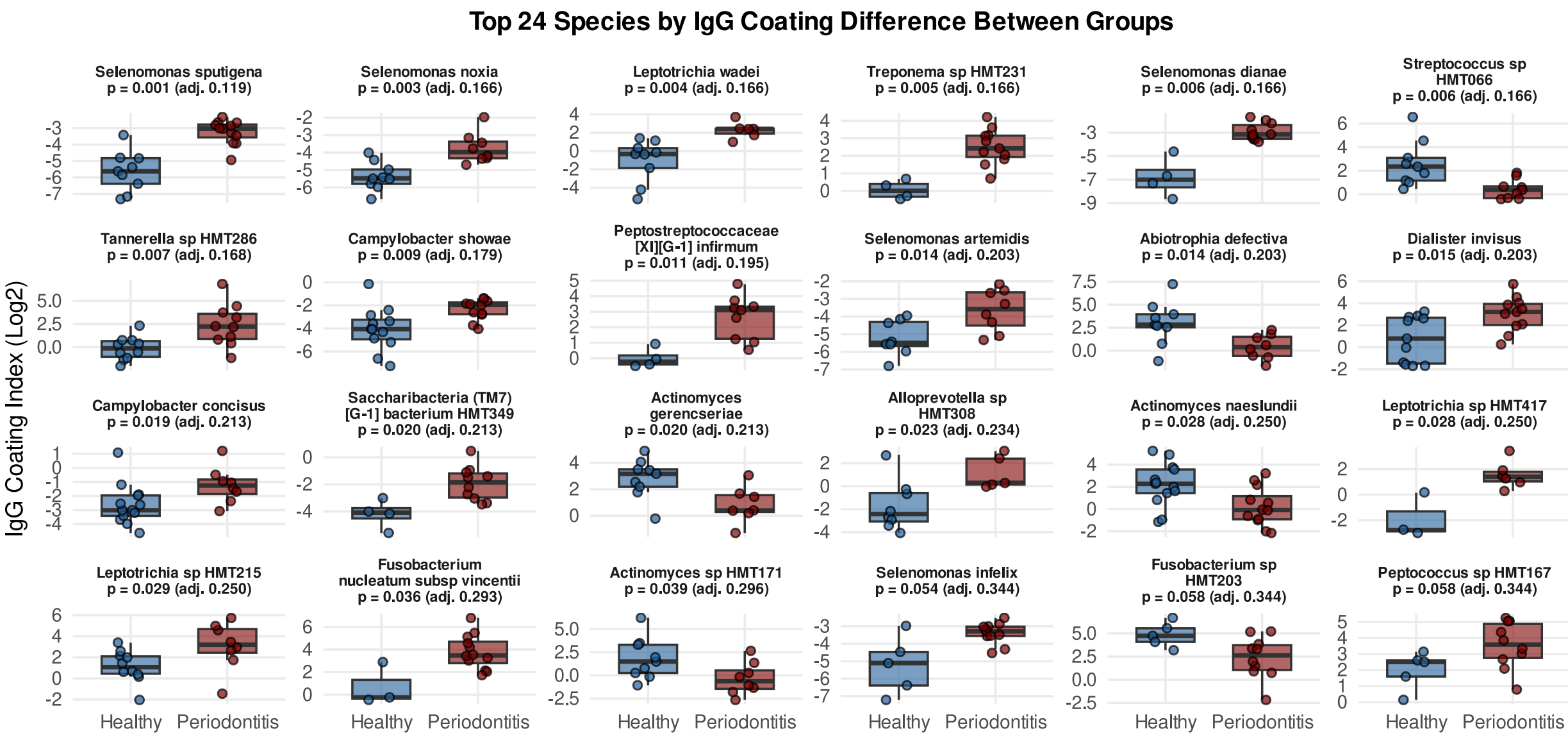

B.

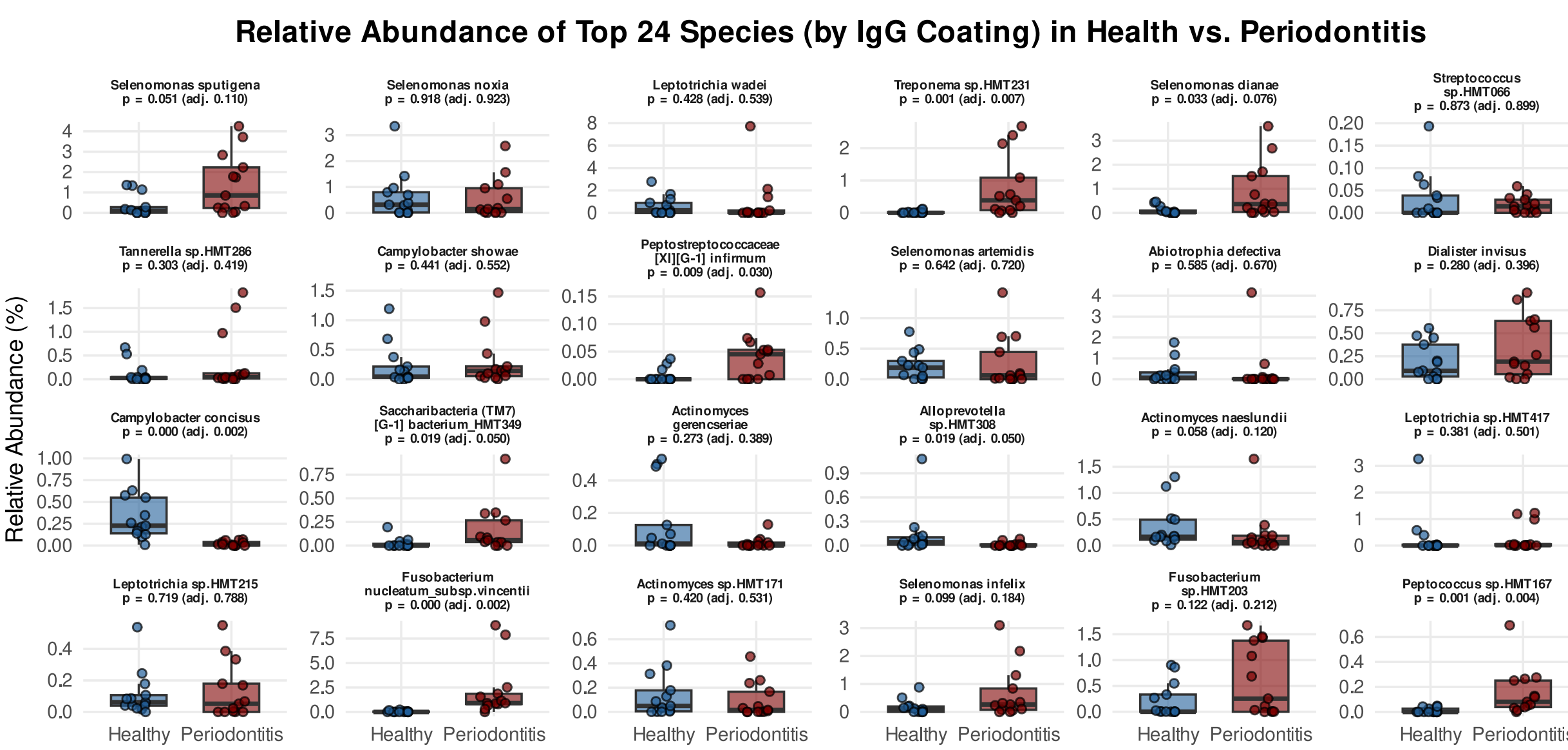

Suppl. Figure 4

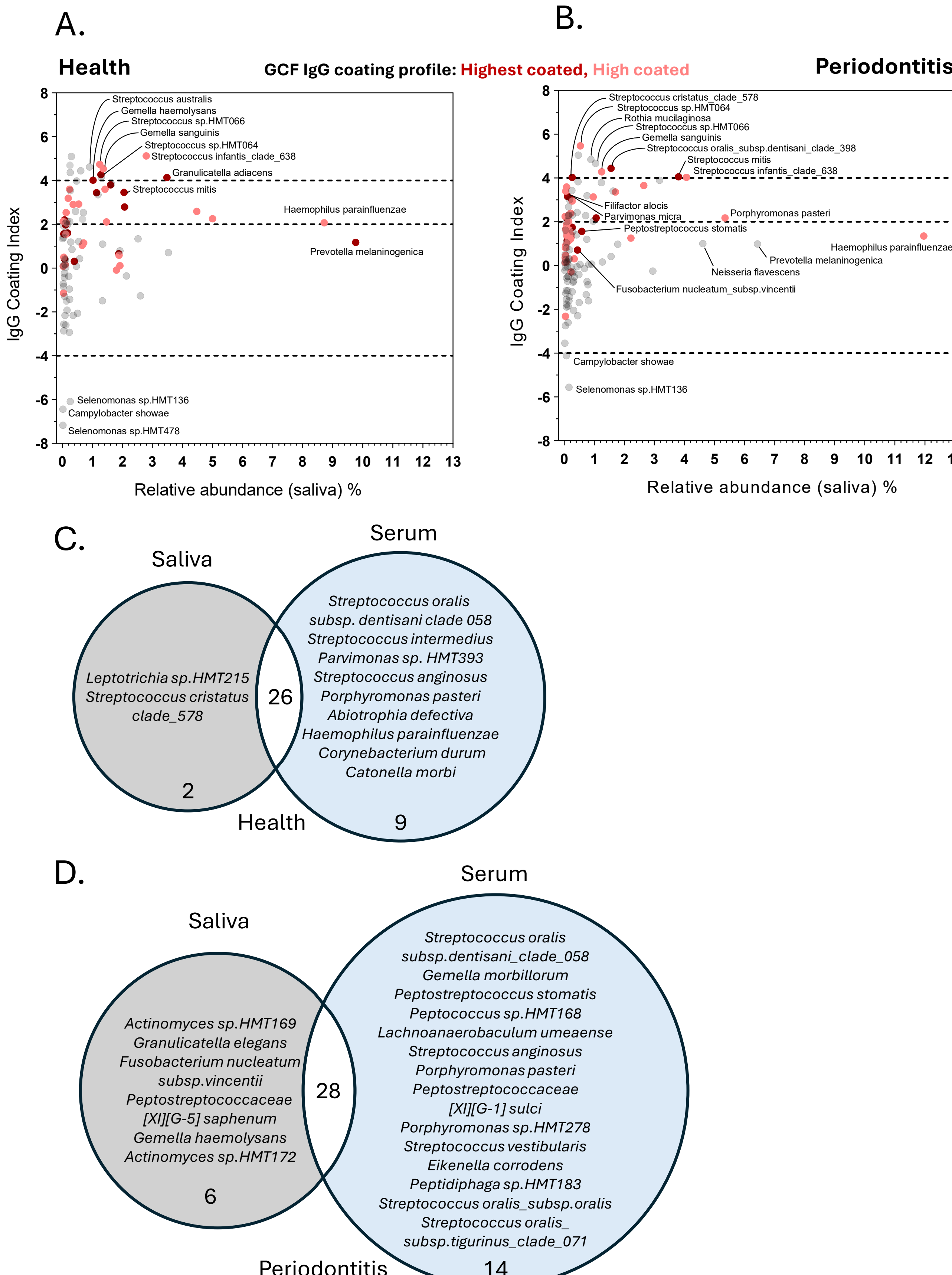

Suppl. Figure 5
